# Real-life experience with inotersen at CEPARM, Hospital Universitário Clementino Fraga Filho, Universidade Federal do Rio de Janeiro

**DOI:** 10.1055/s-0044-1781463

**Published:** 2024-04-05

**Authors:** Moises Dias, Luiz Felipe Pinto, Marcus Vinícius Pinto, Renata Gervais, Paula Accioli, Gabriela Amorim, Mariana Guedes, Carlos Perez Gomes, Roberto Coury Pedrosa, Márcia Waddington-Cruz

**Affiliations:** 1 Universidade Federal do Rio de Janeiro, Hospital Universitário Clementino Fraga Filho, Serviço de Nefrologia, Rio de Janeiro RJ, Brazil.; 2 Universidade Federal do Rio de Janeiro, Hospital Universitário Clementino Fraga Filho, Centro de Estudos em Paramiloidose Antônio Rodrigues de Mello (CEPARM), Rio de Janeiro RJ, Brazil.; 3 Mayo Clinic, Department of Neurology, Rochester MN, United States.; 4 Universidade Federal do Rio de Janeiro, Hospital Universitário Clementino Fraga Filho, Instituto de Cardiologia Edson Saad, Rio de Janeiro RJ, Brazil.; 5 Universidade Federal do Rio de Janeiro, Hospital Universitário Clementino Fraga Filho, Programa de Pós-graduação/Centro de Estudos em Paramiloidose Antônio Rodrigues de Mello (CEPARM), Rio de Janeiro RJ, Brazil.

**Keywords:** Amyloidosis, Peripheral Nervous System Diseases, Amyloid Neuropathies, Familial, Mutation, Missense, Amyloidosis, Hereditary, Transthyretin-Related, Inotersen, Amiloidose, Doenças do Sistema Nervoso Periférico, Neuropatias Amiloides Familiares, Mutação de Sentido Incorreto, Amiloidose Neuropática Hereditária, Inotersena

## Abstract

**Background** Hereditary transthyretin amyloidosis (ATTRv) is an inherited, progressive, and fatal disease still largely underdiagnosed. Mutations in the *transthyretin* ( *TTR* ) gene cause the TTR protein to destabilize, misfold, aggregate, and deposit in body tissues, which makes ATTRv a disease with heterogeneous clinical phenotype.

**Objective** To describe the long-term efficacy and safety of inotersen therapy in patients with ATTRv peripheral neuropathy (ATTRv-PN).

**Methods** Patients who completed the NEURO-TTR pivotal study and the NEURO-TTR OLE open-label extension study migrated to the present study and were followed-up for at least 18 more months to an average of 67 months and up to 76 months since day 1 of the inotersen therapy (D1–first dose of inotersen). Disease progression was evaluated by standard measures.

**Results** Ten ATTRv-PN patients with Val30Met mutation were included. The mean disease duration on D1 was of 3 years, and the mean age of the patients was of 46.8 years. During an additional 18-month follow up, neurological function, based on the Neuropathy Impairment Score and the Polyneuropathy Disability Score, functionality aspects (Karnofsky Performance Status), and nutritional and cardiac aspects were maintained. No new safety signs have been noted.

**Conclusion** The treatment with inotersen was effective and well tolerated for the average of 67 months and up to 76 months. Our results are consistent with those of larger phase-III trials.

## Resumo

**Antecedentes** Amiloidose hereditária por transtirretina (ATTRv) é uma doença hereditária, progressiva e fatal ainda largamente subdiagnosticada. Mutações no gene *transtirretina* ( *TTR* ) promovem desestabilização, desdobramento, agregação e depósito da proteína TTR em tecidos do corpo, o que faz da ATTRv uma doença de fenótipo clínico heterogêneo.

**Objetivo** Descrever a eficácia e segurança da terapia com inotersena no longo prazo em pacientes com neuropatia periférica ATTRv (ATTRv-PN).

**Métodos** Pacientes que completaram o estudo pivotal NEURO-TTR e o estudo de extensão aberta NEURO-TTR OLE migraram para este estudo e foram acompanhados por no mínimo 18 meses adicionais, em média por 67 meses, e por até 76 meses, desde o dia 1 da terapia com inotersena (D1–primeira dose de inotersena). A progressão da doença foi avaliada por medidas padronizadas.

**Resultados** Dez pacientes com ATTRv-PN com mutação Val30Met foram incluídos. A duração média da doença no D1 era de 3 anos, e a média de idade dos pacientes era de 46,8 anos. Durante o período de acompanhamento adicional de 18 meses, a função neurológica, baseada no Neuropathy Impairment Score e no Polyneuropathy Disability Score, os aspectos de funcionalidade (Karnofsky Performance Status), nutricional e cardíacos estavam mantidos. Não se observou nenhum novo sinal de segurança.

**Conclusão** O tratamento com inotersena foi eficaz e bem tolerado por 67 meses em média, e por até 76 meses. Nossos resultados são consistentes com os de estudos maiores de fase III.

## INTRODUCTION

Hereditary transthyretin amyloidosis (ATTRv) is an inherited, severe, progressive, and disabling disease leading to multiorgan failure caused by the systemic accumulation of mutated TTR fibrils into amyloid within tissues, inducing organ damage and ultimately resulting in death in ∼ 10 years if left untreated. 1 2

For many years, liver transplantation was the only treatment for ATTRv as a potential cure for the disease, but studies showed that the results were not satisfactory in certain types of patients such as the elderly, those malnourished, with advanced disease, or with a non-Val30Met mutation. 1 Fortunately, the scenario has changed with the advent of therapies aiming to retard or halt disease progression through gene-silencing strategies, TTR stabilization, and depletion of existing amyloid deposits. 3

Inotersen, a 2'-O-(2-methoxyethyl)-modified second-generation antisense oligonucleotide (ASO), acts by inhibiting the production of mutant TTR by the liver. 4 5 6 By targeting TTR mRNA produced by hepatocytes, it results in TTR mRNA degradation and prevents TTR mRNA translation into TTR protein. 5 The administration of inotersen to human beings is associated with decreases in both the wild-type and mutant forms of TTR. In healthy volunteers, inotersen has shown dose-dependent and sustained reductions in circulating TTR levels of 77% from baseline at a weekly dose of 300 mg after 4 weeks of therapy. 7

The NEURO-TTR 8 was a multicenter, randomized, double-blinded, placebo-controlled, 15-month phase 2/3 trial to determine the efficacy and safety of inotersen in adult patients with hereditary transthyretin-mediated amyloid polyneuropathy. The main inclusion criteria were proven ATTRv peripheral neuropathy (ATTRv-PN) in stages 1 or 2, Neuropathy Impairment Score (NIS) of 10 to 130, and amyloid deposit demonstrated by tissue biopsy. A total of 172 patients received weekly subcutaneous injections of 300 mg of inotersen or placebo. The primary endpoints were change from baseline to week 66 in the modified NIS +7 (mNIS + 7), a validated composite score that includes neurological, neurophysiological, and quantitative sensory testing for different aspects of the autonomic, sensory, and motor neuropathy, and in the total score on the Norfolk Quality of Life – Diabetic Neuropathy (Norfolk QoL-DN) questionnaire After 15 months, significant differences were observed in both primary endpoints in the comparison between the inotersen and placebo groups: the difference in the mNIS + 7 between groups was of -19.7 points (95% confidence interval [95%CI]: -26.4 to -13.0; *p* < 0.001), and of -11.7 points (95%CI: -18.3 to -5.1; *p* < 0.001) for the Norfolk QoL-DN score, favoring the inotersen therapy. Improvement in the mNIS + 7 occurred in 36% of the patients, and in 50% regarding the score on the Norfolk QoL-DN. All prespecified subgroups confirmed a significant benefit of the inotersen therapy. Reductions in circulating TTR in the inotersen group reached steady-state levels by week 13 and were sustained at the nadir of 74% through the end of the intervention period. Safety concerns were mitigated by weekly monitoring of platelets, renal function, and urinary protein excretion. The 3-year open-label extension of the NEURO-TTR OLE trial 9 enrolled 80.1% (113/141) of the patients who completed the pivotal study. Of the 113 patients, 109 entered the OLE study. In total, 70 patients continued to receive inotersen (inotersen-inotersen) and 39 switched from placebo (placebo-inotersen). The inotersen-inotersen group showed sustained benefit in the mNIS + 7, the Norfolk QoL-DN, and in the 36-item Short Form Health Survey, version 2 (SF-36v2), in comparison with the natural estimated evolution of the disease and to the placebo-inotersen group. Patients from the placebo-inotersen group also demonstrated improvement in neurological progression according to the mNIS + 7 compared with the predicted worsening in placebo projected by the NEURO-TTR. The maximum exposure to the therapy was of 6.2 years, and no new safety signals or toxicity were detected.

The aim of the present study is to describe the long-term (efficacy and safety) experience of Centro de Estudos em Paramiloidose Antônio Rodrigues de Mello (CEPARM) with patients continuing to receive inotersen therapy after closure of the main studies.

## METHODS

Patients who completed the Ionis 420915-CS2 (NEURO-TTR) 1 and CS3 (NEURO-TTR OLE) 9 studies were enrolled in the present open-label, noncomparative follow-up study to monitor disease progression by means of the standard measures applied routinely at CEPARM. In the NEURO-TTR trial, 8 139 individuals (81%) successfully concluded the 15-month intervention period. Of the 139 patients, 30 were from South America and Australasia. Of this cohort, 22 patients were recruited in Brazilian sites, with 16 patients from CEPARM. A total o 12 out of the 16 patients completed the double-blinded phase of the trial and were included in the open-label phase. Four patients did not enter the OLE phase due to the following reasons: one death due to criminal asphyxiation; one death due to osteomyelitis-associated sepsis; one glomerulonephritis of unknown cause (the renal biopsy was reviewed by experts, who did not reach a consensus regarding the etiology: inflammatory or underlying disease); and one patient was transferred to another site due to logistic reasons. Brannagan et al. 9 10 published data on the long-term efficacy and safety of inotersen in two different time points: at 2 years, 9 when efficacy and safety data were reported for the whole population (Europe, North America, and Latin America/Australasia), and a 3-year update, 10 in which only efficacy data from European and North American patients were included, totalizing 109 participants because these cohorts completed the study ∼ 9 months before patients from other regions due to a delay in the OLE phase approval process, and the sponsor decided not to further retard the publication.

Of the 12 patients who completed the NEURO-TTR OLE phase, 10 were included in the present postaccess real-life study. Two patients were excluded at the discretion of the investigators: one noncompliant patient for whom an alternative therapy was available; and one patient with liver disease of unknown cause, for whom a clear relationship to the drug could not be established, with vitamin A toxicity being a possibility. For the remaining 10 patients, the following parameters were collected on day 1 of the inotersen therapy (D1–first dose of inotersen in the pivotal and OLE trials) and at the last evaluation (LE; data cutoff in June 2022): Polyneuropathy Disability (PND) score; Coutinho stage (CS) of disease; Neuropathy Impairment Score (NIS); Karnofsky performance status (KPS); creatinine; glomerular filtration rate (GFR); N-terminal pro-brain natriuretic peptide (NT-proBNP); albumin to creatinine ratio; protein to creatinine ratio; Body Mass Index (BMI); presence of cardiomyopathy and heart failure (New York Heart Association [NYHA] class); 12-lead electrocardiogram (ECG); transthoracic echocardiogram (interventricular septum thickness [IVS] and ejection fraction); and amplitude of sural and peroneal compound muscle action potential (CMAP).

The Ethics Committee of the Hospital Universitário Clementino Fraga Filho – Universidade Federal do Rio de Janeiro – approved the additional follow-up of the patients. Informed consent was obtained from all subjects prior to continuing the observation. The Brazilian Health Regulatory Agency (Agência Nacional de Vigilância Sanitária, ANVISA, in Portuguese) approved the study under Special Communication in Clinical Research number 115/2014.

Considering the whole period of therapy from D1 until the LE, the patients received inotersen therapy for an average of 67 months ( Table 1 ).

**Table 1:** Parameters and demographic data of the subjects

Patient	Age at onset of disease (in years)	Time until diagnosis (in months)	Time from disease onset to day 1 of inotersen therapy (in months)	Age at day 1 of inotersen therapy (in years)	Date of day 1	Therapy duration from day 1 until last evaluation* (in months)
1	37†	3	24	39	02/2017	57
2	67	12	24	69	10/2015	73
3	59	12	18	60	11/2015	72
4	34†	24	36	37	10/2016	61
5	34†	18	30	36	11/2015	72
6	35†	24	36	38	07/2015	76
7	39†	48	72	45	04/2017	55
8	31†	12	60	36	10/2016	61
9	38†	24	36	41	11/2015	72
10	65	24	30	67	12/2015	71
	Mean:43.9 ± 13.3	Mean: 20.1 ± 11.6	Mean: 36.6 ± 16	Mean: 46.8 ± 12.6		Mean: 67 ± 7.2

**Notes:** *Last evaluation: June 2022; †Early disease, diagnosed before 50 years of age.

### Statistical analysis

Since the present is a descriptive study, no statistical analysis was planned or performed.

## RESULTS

In total, 10 ATTRv30M subjects (6 males) were included in the present study and followed-up for at least 18 months after leaving the main studies. Two had been previously treated with tafamidis for 18 and 24 months respectively before they were enrolled in the pivotal trial. Six patients had been receiving inotersen since the beginning of the NEURO-TTR trial, while 4 patients started to receive inotersen only in the CS3 (switch from placebo). Thus, D1 means the first day of inotersen therapy either in the NEURO-TTR trial or in the OLE trial; therefore, the duration of the therapy and the time until the LE varied amongst subjects ( Table 1 ). Demographic data are also shown in Table 1 .

**Figure 1:**
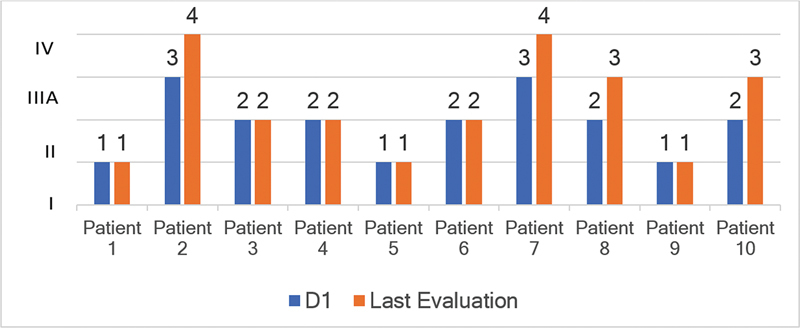
Evolution of individual data from day 1 (D1) through the last evaluation (LE) on the Polyneuropathy Disability (PND) score.

Clinical parameters are shown in Table 2 and graphically in Figures 1 2 3 4 5 .

**Table 2:** Clinical parameters evaluated on day 1 and at the last evaluation

Clinical parameter	Day 1	Last follow-up evaluation (average of 74.5 months)
PND score	3 patients PND I 5 patients PND II 2 patients PND IIIA	3 patients PND I 3 patients PND II; 2 patients PND IIIA 2 patients PND IV
CS of disease	8 patients CS 1 2 patients CS 2	7 patients CS 1 1 patients CS 2 2 patients CS 3
NIS: mean ± SD (range)	48.2 ± 39.8 (12–129.75)	56 ± 40.5 (7–123.5)
KPS: mean ± SD (range)	77 ± 12.5 (50–90)	75.5 ± 18.1 (40–90)
BMI in kg/m ^2^ : mean ± SD (range)	22.6 ± 8.3 (14–30.5)	23.7 ± 5.4 (15.2–31.2)
Cardiomyopathy	3	3
Heart failure	2	2
NYHA class	1 NYHA I/1 NYHA II	2 NYHA I

**Abbreviations:** BMI, Body Mass Index; CS, Coutinho Stage; KPS, Karnofsky Performance Status; NIS, Neuropathy Impairment Score; NYHA, New York Heart Association; PND, Polyneuropathy Disability; SD, standard deviation.

**Figure 2:**
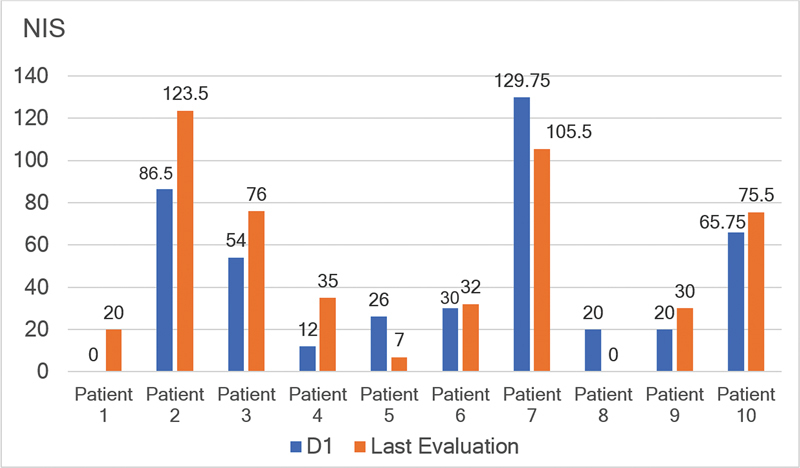
Evolution of individual data from D1 through the LE on the Neuropathy Impairment Score (NIS). Note: D1 from Patient #1 is not available due to missing data; the last NIS evaluation of Patient #8 is not available due to a remote visit.

**Figure 3:**
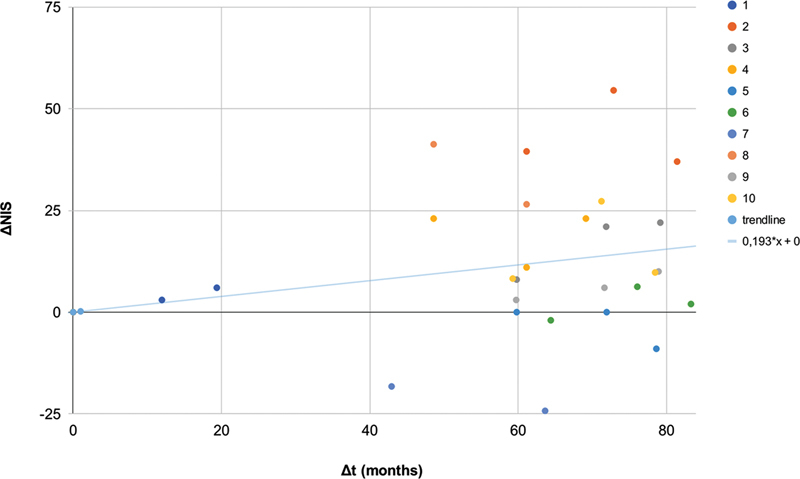
Delta of NIS and NIS trendline.

**Figure 4:**
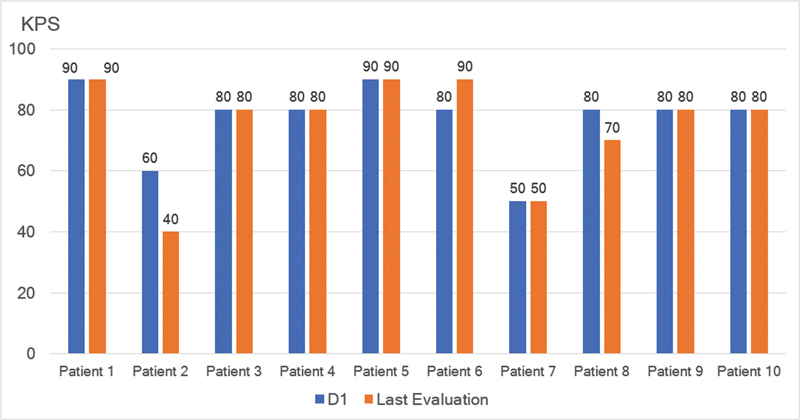
Evolution of individual data from D1 through the LE last evaluation regarding the Karnofsky Performance Status (KPS).

**Figure 5:**
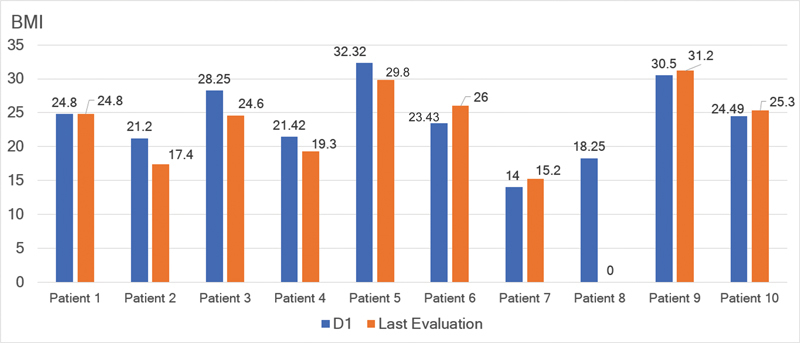
Individual data from D1 through the LE regarding the Body Mass Index (BMI). Note: The LE of Patient #8is not available due to a remote visit.

Three out of 9 patients had an NIS progression of > 10 points and, out of these 3 patients, 2 had NIS > 50 on D1, late-onset, and mixed phenotype (polyneuropathy and cardiomyopathy manifestations).

### Other clinical parameters

#### *Nerve conduction studies*

Nerve conduction studies were compared between the first visit and the LE. The amplitude of the peroneal CMAP was not obtainable in four patients on D1 and in six patients at the LE. The amplitude of sensory nerve action potential of the sural nerve was not obtainable in seven out of nine patients on D1 and in eight out of nine patients at the LE.

#### *Electrocardiogram*

On D1, one patient had normal ECG; two patients had first-degree atrioventricular (AV) block; one patient had premature beats; one patient had nonsignificant ST changes; two patients had right bundle branch block (RBBB) and first-degree AV block and left bundle branch block (LBBB) and first degree AV block respectively; one had LBBB; one had LBBB + qS pattern from V1 through V3 derivation; and one patient had first-degree AV block and atrial flutter. During the long-term follow up, two patients underwent a pacemaker implantation.

#### *Transthoracic echocardiogram*

Interventricular septum thickness > 12 mm was present in 4 out of 10 patients on D1 and in 2 out of 10 patients in the last echocardiogram.

#### *Renal function and thrombocytopenia*

Renal function and protein excretion remained stable. Only 1 patient had a GFR < 60 mL/min/1.73 m ^2^ on D1 and on month 12 of the evaluation. No case of glomerulonephritis or grade-4 platelet decrease was detected. In total, 3 patients had to pause drug administration for 5, 2, and 1 month respectively during the long-term follow-up due to low-grade thrombocytopenia. One patient developed thrombocytopenia due to portal hypertension of unknown cause before data cutoff in June 2022. All of them were able to resume the therapy with inotersen.

## DISCUSSION

Hereditary TTR amyloidosis is a rare, progressive, and debilitating disease, the most common presentation being polyneuropathy. Due to the relatively recent worldwide approval of inotersen, there are very few real-life studies published so far. In Brazil, Tegsedi (PTC Farmacêutica do Brasil LTDA, São Paulo, SP) was approved in 2019, ^6^ and despite not being available in the national formulary, we proceeded treating patients from the Brazilian cohort of the NEURO-TTR pivotal study and OLE trial due to a collaboration with PTC Farmacêutica do Brasil LTDA and Ionis Pharmaceuticals.

Luigetti et al. 11 published data from an early access an inotersen program in Italy. This was a multicenter, observational, retrospective study of ATTRv patients whose primary endpoint was safety. The secondary endpoints were changes from baseline in polyneuropathy stage, PND score, NIS, compound autonomic dysfunction test, Norfolk QoL-DN, troponin, NT-proBNP, interventricular septum thickness, and BMI. A sample of 23 patients was followed up for 14.6 ± 5.9 (range: 6 to 24) months. The patients were treated within a compassionate use program. No patient was discontinued permanently due to thrombocytopenia, but in seven patients, the dosing frequency had to be reduced due to recurrent thrombocytopenia. Only 2 patients progressed, and 21 were stable until the last available assessment. The authors 11 concluded that the long-term safety of inotersen is favorable and that neurologic disease severity at the initiation of therapy is a key factor associated with progression.

The current study study presents the advantage of having a single evaluator performing all assessments consistently throughout the entire study period. Although the study offers valuable information about the effectiveness and safety of inotersen therapy in ATTRv-PN patients over the long term, it is important to keep in mind its limitations. These include a small sample size, absence of a control group, the nonrandomized design, the presence of missing data, the limited duration of the follow-up, and a narrow range of outcome measures.

The cohort represents patients who have transitioned from the controlled environment of the main study to the dynamic setting of real-world circumstances, and the strength of our study comes from the fact that, despite the difficulties imposed by the COVID-19 pandemic, we continued to perform visits and assessments, adding a more realistic perspective.

We conclude that treatment with inotersen was effective and well tolerated for an average of 67 months and up to 76 months of the inotersen therapy. Although no new safety signal was detected, platelet and renal function monitoring is paramount to maintain the safety of the patients. Our results are consistent with those of larger phase-III trials. Further studies with larger sample sizes, longer follow-up periods, and comprehensive outcome assessments are needed to confirm and expand upon these findings.
